# Provenance-Based Trust-Aware Requirements Engineering Framework for Self-Adaptive Systems

**DOI:** 10.3390/s23104622

**Published:** 2023-05-10

**Authors:** Hyo-Cheol Lee, Seok-Won Lee

**Affiliations:** 1 Department of Computer Engineering, Ajou University, Suwon 16499, Republic of Korea; mytion7@ajou.ac.kr; 2 Department of Artificial Intelligence, Ajou University, Suwon 16499, Republic of Korea

**Keywords:** requirements engineering, goal modeling, trust, provenance, self-adaptive system

## Abstract

With the development of artificial intelligence technology, systems that can actively adapt to their surroundings and cooperate with other systems have become increasingly important. One of the most important factors to consider during the process of cooperation among systems is trust. Trust is a social concept that assumes that cooperation with an object will produce positive results in the direction we intend. Our objectives are to propose a method for defining trust during the requirements engineering phase in the process of developing self-adaptive systems and to define the trust evidence models required to evaluate the defined trust at runtime. To achieve this objective, we propose in this study a provenance-based trust-aware requirement engineering framework for self-adaptive systems. The framework helps system engineers derive the user’s requirements as a trust-aware goal model through analysis of the trust concept in the requirements engineering process. We also propose a provenance-based trust evidence model to evaluate trust and provide a method for defining this model for the target domain. Through the proposed framework, a system engineer can treat trust as a factor emerging from the requirements engineering phase for the self-adaptive system and understand the factors affecting trust using the standardized format.

## 1. Introduction

Today, as artificial intelligence (AI) technologies are increasingly used and highlighted in society, software systems have become AI intensive [1,2,3]. AI-intensive systems are not passive systems that wait and respond to user input but proactive systems that provide appropriate services to the user by adapting to various situations. These types of systems are called self-adaptive systems (SASs) [4]. SASs employ four steps: monitoring, analysis, planning, and execution. An SAS monitors the surrounding situation and environment, analyzes the current problem, plans to resolve the problem, and executes the plan to provide better services to the user. In this process, the most important task is collecting the information to define the current problems facing the system. A single stand-alone system has a limited capability to gather this information; thus, such systems need to cooperate with other systems that have various information [5,6,7].

When SASs cooperate with other systems, they should select the appropriate cooperation partner from among many candidates. The meaning of “appropriate” implies not just whether or not the candidates outwardly provide the necessary information but also whether or not the candidates and the provided information are inherently trustworthy [8]. Even though a candidate might provide the necessary information, using this information could lead to an unsatisfactory consequence or an incorrect and dangerous situation because the provided information is untrustworthy [9]. Trustworthiness is a factor that goes beyond the scope of simply whether the target provides the information; it also refers to the reliability of that information [10,11]. Based on this understanding, in this paper, we define trust as “*the level of belief that a cooperative system will share information and services safely by acting as expected.*” Consequently, to select the appropriate cooperation partner, trust becomes an important consideration [12].

Despite the fact that trust becomes an emerging factor in systems development, there is a lack of methods to effectively analyze and represent trust. This problem statement can be divided into two research problems that we need to address. First, there is a lack of a fundamental understanding of trust and the way to systematically analyze it [13,14]. Because of this limitation, it is difficult to analyze a system with regard to trust. Existing trust-related studies simply claim that trust is important and provide certain models for evaluating it [15,16]. They emphasize the importance of trust but do not consider how to analyze and design a system that should consider trust. It is necessary to have a clear understanding of why trust is important, what it is, and under which circumstances it should be considered [17,18]. The next problem is a lack of a proper modeling method to represent trust and its evidence. Existing trust evidence models are usually domain-targeted or limited to the partial and temporal aspects of trust. However, trust is a complicated and complex attribute; thus, it is necessary to define a new trust evidence model that can extensively analyze and represent the various aspects of trust based on an understanding of it [19].

In this research, we propose a requirements engineering framework for trust-aware SASs as a solution to the research problems analyzed above. This framework consists of two phases: a trust-aware requirements modeling phase and provenance-based trust evaluation phase. The first phase, trust-aware requirements modeling, answers the first research problem. This phase is designed for analyzing what constitutes trust in the requirements engineering process. Based on a systematic analysis of trust, the key characteristics related to trust are derived as a trust-requiring situation [20,21]. Next, we propose a method to derive a trust-aware goal model that represents the requirements that must consider trust. The first phase enables us to present a fundamental understanding of trust and how to apply and represent that understanding in the requirements engineering process. The second phase focuses on describing how to present the trust evidence model for a specific domain. The concept of provenance is applied as a key idea to represent and evaluate trust. Provenance is the emerging concept currently used in many domains and research areas [22]. Among the existing studies, the common definition of provenance is “*information about the history, derivation, origin, or context of an artifact*” [23,24,25]. It is helpful to capture the level of trust because trust can be determined by the accumulated behaviors of a system. At the beginning, based on our definition and understanding of trust, we propose a provenance meta-model to represent a basis for trust that is independent of the specific domain. Subsequently, the domain-specific provenance model is defined from the meta-model by analyzing the circumstances under which the system requires trust in the target domain [26]. In addition, we provide an algorithm to evaluate trust using the provenance model.

This paper consists of the following four sections. Section 2 describes the related work from the perspective of requirements engineering for trust and trust evaluation approaches. In Section 3 and Section 4, we provide a detailed explanation of the proposed approach and case studies in two domains: a crowd navigation system and reviewer verification service. Finally, Section 5 presents our concluding remarks with comments on the direction of future work.

## 2. Related Work

In this section, we divide the related studies into two groups and briefly analyze them. The first group focuses on how existing work analyzes requirements engineering for SAS and trust. The second one focuses on the trust evidence models used to evaluate trust in a system.

### 2.1. Requirements Engineering for SASs and Trust

In this paper, we analyze three related studies regarding requirements engineering for SAS and trust. First, Whittle et al. introduced RELAX, a requirement modeling language for SAS with uncertainty [27]. An SAS needs to adapt to various situations that are unknown and unforeseen. Hence, some situations are uncertain. During the requirements engineering process for SAS, it is necessary to represent and analyze the uncertain features of these situations. RELAX uses fuzzy branching temporal logic and represents not only a description of a specific situation the system may encounter but also a comprehensive description of the changing situation. However, although RELAX can design uncertain requirements for SAS, its representation of trust-related requirements is limited. For trust-related requirements, it is important to capture the origin of the uncertain behavior to evaluate the level of trust, but RELAX focuses on representing the spectrum of the behavior instead.

Next, Ali et al. suggested a self-adaptive quality requirement elicitation process to upgrade legacy systems to SASs [28]. The process considers six aspects: source, stimulus, artifact, environment, response, and response measure. During the upgrade process, it is important to consider the quality attributes related to SAS because the functional requirements are considered to be already satisfied. By analyzing system scenarios, a system engineer determines whether or not the scenario should be self-adaptive. Subsequently, scenarios that should be self-adaptive become self-adaptive quality attribute scenarios based on the six aspects and are used to convert the legacy system into an SAS with new quality attributes. However, this approach focuses on upgrading a legacy system to an SAS; thus, it requires an initial process to analyze and define the system requirements without considering self-adaptation. In addition, although trust is considered to be one of the quality attributes, unlike the other quality attributes, it is difficult to apply this approach to determine trust because trust requires an additional model to express its features and characteristics.

The last study focuses on trust requirements. Grüner et al. analyzed trust requirements in decentralized identity management [29]. Applying blockchain technology and defining the topology patterns, they suggested several trust requirements that should be considered in decentralized environments. Although there are several methods to guarantee identity, it is difficult for a system to guarantee its own identity in decentralized environments because the existing methods require connection to centralized identity providers. To overcome this limitation, they used blockchain technology for the decentralized identity verification process and analyzed the trust requirements to safely ensure the results using the decentralized approach. However, this approach focuses on the identity management domain and is difficult to utilize in another domain. Moreover, it is still in the early stages of trust requirements analysis and lacks a modeling process for the trust requirements.

These studies attempted to analyze the requirements for SAS and trust, but there are still some limitations, particularly concerning trust requirements analysis and its modeling. To overcome these limitations, we propose a trust-aware requirements engineering process. In this process, a trust-aware goal model is derived from the use case model by considering a trust-requiring situation, which is analyzed based on the characteristics of trust. It also can be used in various domains.

### 2.2. Trust Evidence Models for Evaluation

There are several kinds of trust evaluation method. We categorize them into four types according to the trust evidence model: (1) central, (2) direct, (3) indirect, and (4) rule-based methods [30,31,32,33,34,35,36,37].

In the central methods, a central authority system is used to verify whether or not a system is trustworthy. The central authority system manages the information to confirm that the system is trustworthy; that is, the authorization process is considered to be the trust evidence model. To be evaluated as a trustworthy system, it is necessary to register in advance with the required information such as certification issued by a government. However, in open and decentralized environments, it is difficult to implement an omniscient system that can manage all the information about trustworthy systems and queries about the trustworthiness of a certain system.

The direct method utilizes past experiences gathered from the system’s direct interactions with other systems as the trust evidence model. Because the system considers its own experiences, it does not need to interact with other systems to evaluate the trust of a target system using standards and criteria that are not its own. However, the system cannot interact with all possible candidates and is difficult to evaluate trust of a system with which it has had little or no interaction. In addition, because only the system’s own experience is used, objective evaluation is not considered, and it takes a long time to accumulate sufficient data required to evaluate trust.

Unlike the direct method, the indirect method uses other systems’ experiences as the trust evaluation model. In open and decentralized environments, all systems can interact with other systems and accumulate the results of those interactions as experience. When a system evaluates its trust of a specific target system, the system can ask other systems for their experiences with the target system. Thus, this method can be applied even when the system does not have experience with the target system. Moreover, it results in a more objective decision because trust is evaluated from the evidence of various systems. However, the trust evaluation results derived by this method can be manipulated by the organized behavior of malicious systems and groups.

The last method is the rule-based method. It uses predefined rules to determine if the target system is trustworthy. Therefore, an initial process is required to define the rules based on an understanding of the domain and trust by system engineer and domain experts. In the rules, many kinds of information can be utilized, such as system specifications or performance factors. Even though this method is effective for evaluating trust by considering the various aspects of trust, it is difficult to define the appropriate rules for each domain and every circumstance.

In general, these methods are not used alone but in combination with other methods to complement the limitations of each one. For example, a combination of the direct and indirect methods is widely used because it can use the subjective and objective information together. However, even if these methods are combined in various configurations, they have a common limitation in that they only use a simplified score for the trust evaluation process. The simple score is intuitive and easy to use, but it is difficult to capture the diverse aspects of trust. To overcome this limitation, we propose a provenance model as a new type of evidence of trust. The provenance model is a model that represents the self-defining information of a system and its behaviors from its origin to the present. This model has the capability to represent various system features related to trust. Therefore, using provenance model as trust evidence model makes it easy to capture trust-related features.

## 3. Proposed Approach

In this section, we describe the details of the proposed approach. From the two research problems defined in the introduction, we derived the following research questions: (1) What is trust and what are the conditions under which trust is required? (2) What are the necessary grounds for determining a trustworthy system, and how can we model those grounds? To answer the first question, we need to present our fundamental understanding of trust, how to analyze trust in various situations, and how to represent the analysis results. To answer the second question, we must determine the evidence to be used for verifying the system as trustworthy and define the model for the new evidence. The proposed approach can answer these two questions. Based on the analysis of trust, the trust-requiring situation is defined, and trust-aware goal modeling is described by applying the trust-requiring situation to the requirements engineering process. The provenance model is used as a new trust evidence model to evaluate trust, and a modeling method to apply it in specific domains is explained. In addition, the trust evaluation process with the provenance model is described.

**Figure 1:**
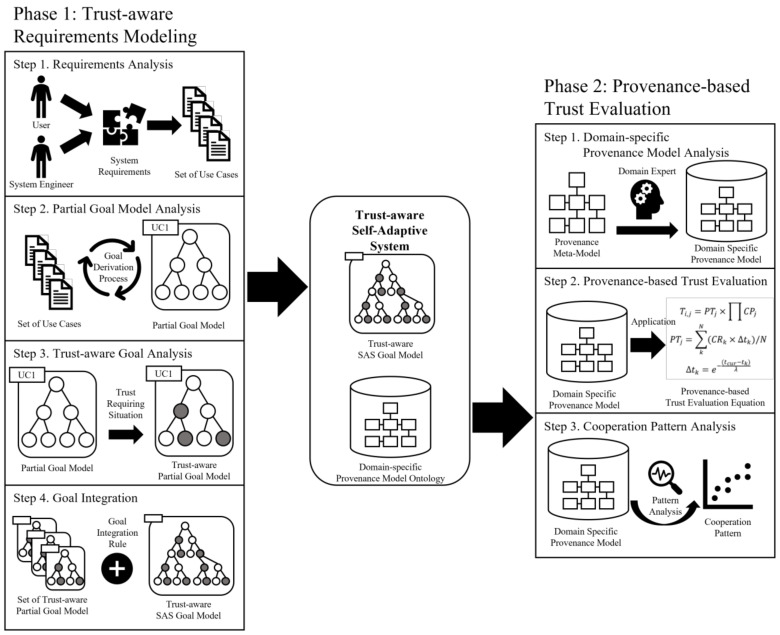
Overview of the proposed framework.

Figure 1 shows an overview of the proposed framework, which consists of two phases. The first phase has four steps and the second has three steps. The first phase is designed for creating a trust-aware goal model. The four steps are as follows: (1) *requirements analysis*, (2) *partial goal model analysis*, (3) *trust-aware goal analysis*, and (4) *goal integration*. In the requirements analysis step, the system functions are analyzed individually to derive a use case model. In the second step, each derived use case model is used to define a partial goal model representing the partial behaviors of the system. In the third step, the trust-aware partial goal model is defined by applying the characteristics of trust to the partial goal model, and in the last step, each trust-aware partial goal model derived from individual functions is integrated into the completed goal model for the entire system.

The second phase is designed for creating the provenance modeling and trust evaluation methods using the provenance model. It consists of three steps: (1) *domain-specific provenance model analysis*, (2) *provenance-based trust evaluation*, and (3) *cooperation pattern analysis*. In the domain-specific provenance model analysis step, the provenance meta-model is defined based on the understanding of trust analyzed in the first phase, and a domain-specific provenance model suitable for each domain is analyzed using the meta-model. Next, the analyzed model is conceptualized and stored as an ontology model. In steps 2 and 3, the trust evaluation is performed from different viewpoints, and they are combined into the system trust value. The details of each phase and step are described in the following subsections.

### 3.1. Phase 1: Trust-Aware Requirements Modeling

**Figure 2:**
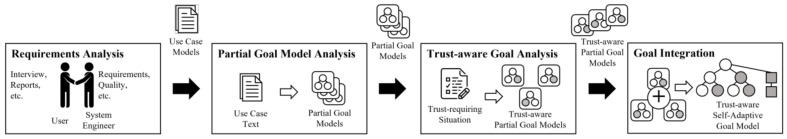
Trust-aware requirements modeling process.

In this subsection, we describe the details of the first phase. Figure 2 shows the trust-aware requirements modeling process. Each step has unique artifacts as the output that are passed to the next step as the input. A description of each step, including the process and the artifact, is presented in the following.

#### 3.1.1. Step 1: Requirements Analysis

The first step is the requirements analysis, which is the process of collecting the requirements that the user expected from the system and representing the collected requirements with a use case model. There are various methods to analyze and represent requirements; we apply the use case model, which is a user-friendly approach. Because the use case model captures the requirements with the scenario-based approach, it is easy to collect the required information from the user and represent it in a user-friendly perspective. Many templates for the use case model exist. Among them, we adopt the template suggested by Cockburn and modify it slightly according to our purpose [38].

Table 1 presents the modified use case model template and an explanation of each element. Basically, we adopt the template of Cockburn and remove the elements that are unused or unimportant for the proposed approach such as the priority or the frequency elements. The main purpose of adopting the use case model is that it is easy to analyze the system scenario and the relationship with other use cases; thus, unnecessary elements are removed for simplicity of analysis. Instead, we add the *required information* element. Required information, as the name suggests, is an element that represents information necessary for the operation of the corresponding function in the use case scenario. It is essential for analyzing the trust-aware requirements.

Using this template, a system engineer analyzes each system function with respect to the use case model. During the analysis, we focus on the objective of each function and the scenario for the operation. The use case model enables us to analyze the requirements in a user-friendly form, and it is easy to obtain mutual understanding and agreement, which makes it possible to derive and model the thoroughly analyzed requirements.

#### 3.1.2. Step 2: Partial Goal Model Analysis

The second step is the partial goal model analysis step. In this step, the system engineer derives the goal model from each use case. Because the derived goal model focuses on the specific function, we call this goal model the partial goal model. In the previous step, we focus on the requirements modeling using a user-centric perspective. In this step, we focus on the requirements modeling from an engineer-centric perspective; that is, the modeling perspective is changing from the user to the engineer. This helps the system engineer appropriately understand the user’s needs and translate them into a modeling method specific to the users. Among the various modeling approaches, we adopt a goal modeling method. Using this method enables us to analyze the correlation of various stakeholders involved in the system operation and convert them from abstract concepts to actual units of action in the system.

To help the system engineer derive the partial goal model from the use case model, we suggest three derivation processes: (1) a three-layer goal derivation process, (2) an extension goal derivation process, and (3) a goal relation derivation process. The first suggested process is the three-layer goal derivation process. The aim of this process is to derive goal instances from the use case scenario using three layers: (1) the use case goal layer, (2) the actor goal layer, and (3) the system behavior goal layer. We separate the three layers because they represent different perspectives. The use case goal layer includes the main objective for the entire use case. Thus, it comprises the higher-level goal instances in the goal model. In the use case template, the use case goal instance can be derived from the *goal in context* element. Next, the actor goal layer addresses the user’s behavior for achieving the use case objective. The user is represented as the *main actor* element in the use case template. The user’s behaviors in the *description* and the *extension* of the use case templates become actor goal instances. As a result of the user’s behaviors, we can expect the system to react, and this forms the system behavior goal in the third layer. The system’s reactions are also represented in the description and the extension of the use case templates. Thus, there are fundamentally hierarchical relationships between goal instances in each layer. A set of system behavior goals is required to achieve the actor goal, and a set of actor goals is required to achieve the use case goal.

The next process is the extension goal derivation process. This process creates an abstract goal instance as the upper-level goal instance by analyzing the points of variation in the use case scenario. In the use case template, there are subscenarios that are extensions of the main success scenario. By analyzing this scenario, we can determine the common objective among the several extension behaviors. For example, in a navigation system, there are many options for determining the best route, such as the shortest route with higher tolls or the cheapest route with the longer distance. Each option has a unique process for calculating the route, but they have a common objective, which is to provide the best route. Because this common objective is not directly represented in the scenario, we suggest defining the extension goal instance in the proposed approach. Defining the extension goal instance helps the system engineer specify a group with related requirements and design detailed system architectures in the system design process.

The last process is the goal relation derivation process, which aims to analyze the specific relationships between derived goal instances. The goal model usually consists of the group of goal instances and the relationships between them. Because we focused on deriving goal instances in the previous processes, this process focuses on analyzing the relationship between goal instances. In this process, we consider two types of relationships: AND and XOR. An AND relationship indicates that all subgoal instances should be satisfied to achieve the upper-level goal instance. Thus, it can apply to the connections between different layers, such as the use case goal and actor goals or the actor goal and system behavior goals. An XOR relationship indicates that only one subgoal instance can be satisfied to achieve the upper-level goal instance. It can apply to the extension goal instance and its subgoal instances because only one of them can be satisfied.

In this step, system engineer can derive a set of the partial goal model corresponding to each use cases of the system. This means that the requirements for each function are analyzed individually, and it helps separate the analysis of the purpose and behavior of the system for each function. By feeding this information to the next step, it is possible to analyze the trust in individual functions rather than analyze the trust for complex behavior.

#### 3.1.3. Step 3: Trust-Aware Goal Analysis

The next step is the trust-aware goal analysis step, which is the most important step in the first phase. By applying trust-aware goal analysis to the partial goal model derived in the previous step, general goal instances for which trust should be considered are converted into trust-aware goal instances, and the partial goal model becomes the trust-aware partial goal model. Therefore, it is necessary to define guidelines for determining the situations in which trust is required by analyzing the characteristics of trust. We define a trust-requiring situation using three criteria to define trust-aware goal instances and the trust-aware partial goal model.

However, we should first understand what trust is. In this research, trust is defined as “the level of belief that a cooperative system will share information and services safely by acting as expected.” From this definition, we can elicit three criteria associated with trust; that is, the situation must be (1) *informative*, (2) *interactive*, and (3) *arbitrary*. From these three criteria, the trust-required situation is defined to determine when trust should be considered. The system engineer applies the trust-required situation to convert a legacy goal instance into trust-aware goal instances. The following text details how to apply each criterion.

First, the informative criterion states that trust should be considered when specific information is required in a scenario to understand the problem and derive an appropriate solution because it is directly related to trust of the information or the information provider. That is, there must be an object for which trust is to be evaluated or an element that evaluates trust. The informative criterion can be evaluated as the first step for checking a trust-requiring situation by using the required information elements of the proposed use case model. This element was devised to analyze and represent which information is used in the specific step of the use case scenario. Therefore, the informative criterion is satisfied when the acquisition of certain information is necessary, such as for monitoring the environment or checking the current state of the system.

Next, the interactive criterion indicates that trust should be considered when the system is interacting with other systems. If the system is a stand-alone system, its ecosystem is limited and independent of other systems. This means that the system does not have any cooperation partners, and trust does not need to be considered. However, a single system has a limited ability to gather the information required for decision-making processes. This encourages a system to interact with other systems to gather the appropriate information. Therefore, the interactive criterion is satisfied when the system requires information that it is unable to acquire by itself, and this can be verified by checking whether or not the information is available by itself in the use case scenario and system specification.

The last criterion is the arbitrary criterion, which indicates that the system should be cooperating with arbitrary systems. When the system is interacting with a well-known system such as a public or government system, it does not need to determine whether or not the cooperation partner is trustworthy. These systems are considered to be trustworthy because they are focused on the public good and controlled by the government. However, in socio-technical environments, a system should interact with unknown systems without any biased background knowledge. Unknown systems could provide incorrect information or low-quality services, leading to trust-related issues. Thus, the system must verify the trustworthiness of the unknown system in advance. To verify the arbitrary criterion, the system engineer can check whether authorized information providers are involved in the cooperation based on the characteristics of the information. Consequently, the goal instances that satisfy these three criteria are converted into trust-aware goal instances.

In this step, we analyze the concept of trust and subsequently suggest the guidelines for defining a trust-aware goal instance as a trust-requiring situation. These guidelines help the system engineer to convert a partial goal model with general goal instances into a trust-aware partial goal model with trust-aware goal instances.

#### 3.1.4. Step 4: Goal Integration

The last step of the first phase is the goal integration step. In the previous step, we focused on deriving the trust-aware partial goal model for individual functions. After which, we need to integrate these models into the trust-aware system goal model representing the overall system goal.

If all trust-aware partial goal models are separate and distinct, goal integration is not necessary because it has already been completed by deriving each model. However, even though each goal model is derived from different functionalities, most of them are interlinked and related to each other. In this step, to provide a way to combine the trust-aware partial goal models as a trust-aware system goal model, we propose three goal integration rules: (1) the *actor grouping rule*, (2) the *explicit integration rule*, and (3) the *implicit integration rule*. When these rules are applied, the elements of the use case model are used because they contain information about the relevance to other use cases.

The first rule is the actor grouping rule, which integrates the trust-aware partial goal models into the goal model of a specific stakeholder. If the trust-aware partial goal models target the same stakeholder, they will eventually focus on satisfying that stakeholder’s objective. Therefore, they can be used as the subgoal model of the stakeholder’s fundamental goal. This rule can be verified by the main actor element in the use case model. After the system engineer has applied this rule, there will be several groups of trust-aware partial goal models depending on the main actor.

The next rule is the explicit integration rule, which connects the trust-aware partial goal models with the hierarchical relationships using the superordinate and subordinate elements in the use case model. Both elements show the relationships within the use case model. If the use case model includes a complicated step to describe the scenario, we can replace the complicated step into the subordinate use case, and the original use case model becomes the superordinate use case model. Using this rule, the system engineer can derive the hierarchically connected goal models and design a preliminary skeleton for the goal model of each actor. However, this approach has a limited ability to integrate all trust-aware partial goal models because the superordinate and subordinate are optional elements, and they only describe a hierarchical relationship without sequential relationships and specific connected points.

To supplement the explicit integration rule and recognize further relationships, we added the implicit integration rule at the conclusion of the goal integration step. This rule uses the *precondition*, *end condition*, and *trigger* elements of the use case model. They, respectively, indicate the prerequisites needed to execute the use case, the expected statement after the use case has been executed, and the event that causes the use case to execute. In contrast to the *superordinate* and *subordinate* elements, these three elements are mandatory. They do not clearly explain the specific connection with other use cases but instead provide the evidence required to discover sequential relationships with other use cases. Commonly, relationships occur between the *end condition* element and the *precondition* or *trigger* elements because the end of a certain use case brings about the start of other use cases. Using them, system engineers can analyze and derive the sequential relationship between the use case models and the connected points between goal instances.

These three integration rules are used to connect the trust-aware partial goal models that were individually derived. Consequently, this step results in the integrated trust-aware goal model for the entire system. In this process, connections are made that consider the user aspect, functional hierarchical relationships, and priority relationships. Through this integrated goal model, system engineers can consider trust in the process of system development and systematically analyze how the behavior of the system affects trust.

### 3.2. Phase 2: Provenance-Based Trust Evaluation

Because the first phase focuses on analyzing the trust concept in the requirements engineering process, the second phase focuses on defining a model to represent and utilize the analyzed trust. That is, we need to define a new trust evidence model for evaluating and utilizing trust.

For this phase, we propose the provenance modeling method and provenance-based trust evaluation method. Provenance is currently used in many domains and research areas. Among the existing studies, a common definition of provenance is “information about the history, derivation, origin, or context of an artifact.” Based on this understanding, provenance can be used as novel evidence of trustworthiness because trustworthiness can be determined from the accumulated behaviors of a system. In addition, existing trust evaluation models have evaluated the trustworthiness of a system through the fragmentary perspective of the system, and this makes difficult to understand the rationale behind the trust evaluation results. Because the provenance model considers not only the fragmentary behaviors of the system but also the correlation and connectivity between these behaviors, it is possible to effectively assess trustworthiness and analyze the meaning of how the trust value was derived in the process of evaluating trust.

#### 3.2.1. Step 1: Provenance Model Analysis

First, we propose a provenance model as the trust evidence model to be used for evaluating whether or not a system is trustworthy when the trust-aware goal instances have been activated. Provenance models are widely adopted in various research areas as the data representation model for build assessments about data reliability and quality [39,40,41,42]. However, because the existing provenance models focus on data or are specialized for specific domains, it is difficult to apply them to other domains that were not initially targeted [43,44,45]. Therefore, in this research, we define a provenance meta-model, which is a general model that can be adapted for a specific target domain. Using the provenance meta-model, the system engineer applies their understanding of the target domain and the system to be evaluated for trustworthiness to define the domain-specific provenance model.

**Figure 3:**
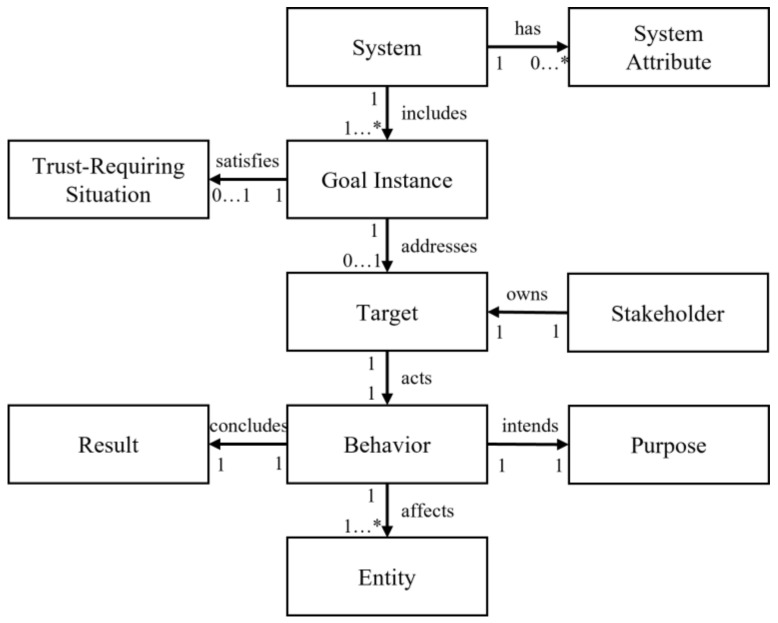
Provenance meta-model.

Figure 3 shows the proposed provenance meta-model. It consists of 10 elements with various relationships. This meta-model was inspired by PROV-DM, which is a conceptual data model designed by the W3C provenance group. The first element in the meta-model is the *system*, which is a unique element that represents the system itself. It has some *system attribute* elements to specify the system features, such as the owner or statement. The system includes many *goal instance* elements. Some of them satisfy the *trust-requiring situation* element and become the trust-aware goal instances. The trust-aware goal instances address the *target* element, which triggers and activates the trust-aware goal instance. It is owned by *stakeholder* elements related to the activated goal instance or the target. To activate the goal instance, the target performs a certain action, which is represented as the *behavior* element. The behavior element has a *purpose* intended by the target, *entity* affected by the action, and *result* concluded by the action.

Using the proposed provenance meta-model, the system engineer can define the domain-specific provenance model by specifying the elements of each concept. The domain-specific provenance model can capture the trust-related behaviors of the specific domain; thus, it can be used as trust evidence to evaluate system trust with a comprehensive understanding of the target domain and trust. When the system engineer defines the domain-specific provenance model, the use case model and trust-aware partial goal model can be used to determine the elements of each concept because they include all system behaviors and trust-related characteristics.

The domain-specific provenance model is defined as an ontology that can accumulate huge amounts of data and represent the relationships among data for inference. An ontology is a model that defines the conceptual elements of a specific domain in a structure that can be understood by the system. By adding actual instances to the defined concept, it becomes possible to infer the relationships or hidden meanings between instances, and it helps to effectively build and manage the knowledge base. That is, the provenance meta-model becomes the class of the upper concept, and the domain-specific provenance model is defined as the class of the lower concept. Subsequently, at runtime, actual information is accumulated as the instances for the defined classes, and the system uses them to evaluate the level of trust by inferring the relationships among them.

There are some advantages to using the provenance model as the trust evidence model. One is that the provenance model is able to represent the system’s various behaviors and perspectives from the origin of the system to the current status. This is helpful for understanding the system’s history and intention, which are important for determining if the system is trustworthy. In addition, the provenance model represents not only the unidimensional perspective of the target systems but also the rationale behind the system’s behaviors. The provenance model contains the group of behaviors and the relationships among them. By analyzing the patterns in the relationships, the cooperation pattern can be defined to understand and apply the social aspects of trust for the trust evaluation. Among the huge amount of information in the provenance model, there are the hidden and concealed behaviors, and we analyze them as the cooperation patterns that affect the level of trust. A single behavior does not reveal any intentions of the system; however, a set of behaviors may reveal the rationale behind the system and certain indications that increase or decrease the trustworthiness of a system. In the following steps, we describe how the provenance model can be used to evaluate trust in detail.

#### 3.2.2. Step 2: Provenance-Based Trust Evaluation

A single provenance model only represents the fragmentary snapshots of a system. Thus, we need an algorithm to analyze a set of provenance models and derive quantitative results. In this step, we suggest a trust evaluation algorithm that focuses on provenance-based trust, which is expressed as follows.

(1) $$T_{i,j}={\mathrm{PT}}_{j}\times {\mathrm{CP}}_{j}$$

(2) $${\mathrm{PT}}_{j}=\overset{N}{\underset{k}{\sum }}\left({\mathrm{TR}}_{k}\times \Delta t_{k}\right)/N$$

(3) $$\Delta t_{k}=e^{-\left(t_{\mathrm{cur}}-t_{k}\right)\lambda }$$

In Equation (1), $T_{i,j}$ denotes the trustworthiness of the j-th system as determined by the i-th system. It is calculated by multiplying the provenance-based trust evaluation of the j-th system (${\mathrm{PT}}_{j}$), which intuitively evaluates reliability using the provenance model itself, and the cooperation pattern for the j-th system (${\mathrm{CP}}_{j}$), which analyzes the characteristic patterns between models to identify hidden intentions and meanings. In this step, we focus on the provenance-based trust evaluation part.

Provenance-based trust is calculated according to Equation (2). This equation is the sum of the products between the trust result value for the k-th provenance model (${\mathrm{TR}}_{k}$) and the function ($\Delta t_{k}$). This is because, depending on the domain, the result can be represented as a numeric value or the categorical value, and the system engineer may need to replace the categorical result with a numeric one. For example, if the result is defined as a “Success” or “Failure,” we can replace “Success” with 1 and “Failure” with 0. Next, $\Delta t_{k}$ is a function to reduce the influence of the trust results over time for k-th provenance model. Even though two results have same trust results, according to the time when the corresponding provenance model was generated, the result in the time close to the present has a greater influence than the result in the past. Equation (3) shows the detail of $\Delta t_{k}$. It is a formula for the half-life of an exponential decay. In this equation, λ is the decay constant, which indicates the time sensitivity of the trust value. That is, the larger the decay constant, the more quickly the trust results decrease over time. The decay constant may be defined differently according to the domain because different domains have different temporal perspectives when considering trust.

Provenance-based trust focuses on information that is directly visible in the provenance model. It is difficult to understand and reveal the hidden intentions and real purpose of a system behind its rationale. To overcome this limitation, we also suggest cooperation pattern analysis, which is the next step.

#### 3.2.3. Step 3: Cooperation Pattern Analysis

In this section, we explain the cooperation pattern. When the system evaluates the trustworthiness of other systems, it is important to understand the hidden intentions of the system, which are difficult to discover using the provenance-based trust. In other words, it is important not to define only the fragmentary appearance of each provenance model like a dot but to connect the dots to the line and grasp one big flow and intention. From this perspective, it is necessary to identify the patterns among the accumulated provenance models, analyze the meanings of the patterns, and consider them in the trust evaluation of the system.

The cooperation pattern consists of four elements: (1) *name*, (2) *type*, (3) *discriminant equation*, and (4) *influence equation*. The *name* is an element that describes the characteristics of the pattern and reveals the meaning of the pattern. The *type* element consists of incentives and penalties, and each indicates whether the corresponding pattern is a type that increases trust or a type that decreases trust. Next, the *discriminant equation* is used as a criterion for determining whether or not a corresponding pattern can be applied to each trust evaluation target. Lastly, the *influence equation* determines the degree of increase or decrease in trust towards the target to which the pattern has been applied through the discriminant equation. The degree of increase or decrease in trust is defined differently depending on the meaning and influence of each pattern.

(4) $${\mathrm{CP}}_{j}=\prod {\mathrm{IE}}_{i}$$

Equation (4) describes how the cooperation pattern is applied. Here, ${\mathrm{IE}}_{i}$ is the influence equation value of the i-th applicable pattern, and ${\mathrm{CP}}_{j}$ denotes the product of the influence equation values of all applicable patterns for the j-th system. During the trust evaluation process, more than one pattern can applied to a system; that is, multiple patterns can be applied simultaneously. The product of the influence equation values of all applicable patterns is then multiplied by the provenance-based trust in Equation (1). By combining the results of the cooperation patterns with the provenance-based trust, it is possible to evaluate a more realistic trust that reflects not only the directly observed actions of the target system but also the intentions and purposes of the target system that are not expressed outwardly. Finally, using the trust results derived in the proposed algorithm, trustworthy targets can be selected. The system cooperates with these targets, and this cooperation is accumulated to generate a new provenance model.

In this section, we presented two aspects of the proposed approach: the trust-aware goal modeling process and the new trust evaluation algorithm with the provenance model. Whereas existing methods focus mainly on the trust evaluation process, the proposed approach helps to analyze and understand what that trust comprises in the requirements engineering stage. In detail, trust can be considered in the overall process of system development by designing a trust-aware goal model and using it in the system development process. By analyzing the characteristics of trust, we define the criteria to verify whether trust should be considered in given requirements or not. In addition, we designed a provenance model as a new trust evaluation model. Using the proposed provenance meta-model, the system engineer can design a domain-specific provenance model that is able to represent the various behaviors of the system. That is, analyzing the accumulated provenance model can help the system understand which behavior can be used to evaluate the system trust. Using the provenance model with the cooperation patterns, the system can consider the intentions and hidden purposes of a target system and quantify its trustworthiness. As a result, the proposed approach allows the system engineer to analyze trust from new and diverse perspectives. Rather than simply evaluating trust, a more realistic and semantic trust evaluation can be performed by identifying the meaning of trust and situations in which trust is required and linking it to the trust evidence model and trust evaluation process. Consequently, in practice, the proposed approach is applied to the requirements engineering stages for identifying the trust-aware goal model with a comprehensive understanding of trust and deriving the trust evidence model to recognize the various aspects of trust as a provenance model. It helps the system engineer to consider trust and a provenance model in the process of system design and development later.

## 4. Case Study Design

In this section, we describe the design of the case studies that were conducted to verify the feasibility and the efficiency of the proposed approach. The verification process was developed based on a case study design methodology that includes a theoretical evaluation and empirical evaluation [46]. In the first step, the study questions should be derived from the corresponding research question. The study questions indicate why and how the proposed approach addresses the research questions. The study questions are specified in the study propositions, which consist of the hypotheses to be accepted using various evidence which can be used to claim that the study questions have been resolved. The evidence is defined as the unit of analysis and is collected through the theoretical evaluation and empirical evaluation processes. In the theoretical evaluation, we apply the proposed approach in two application domains and examine the generated artifacts. By contrast, in the empirical evaluation, we explained the proposed approach to three subject matter experts (SMEs). We then examined how they used the proposed approach and what results were derived. Furthermore, we asked them to assess the proposed approach from their experience with the case study and compare it with the legacy approach.

For the theoretical evaluation, we selected two application domains: a domain in which trust is analyzed from a collective point of view and another in which trust is analyzed from an individual point of view. We performed this because trust pursued by a specific group differs from trust pursued by individuals. First, the crowd navigation system for unmanned vehicles (CrowdNav-UV) is used to demonstrate trust from a collective point of view. The CrowdNav-UV system is a navigation system for unmanned vehicles that aims to provide better navigation services while sharing road conditions and additional information about each vehicle [47]. During the navigation process, it is important to cooperate with trustworthy systems because if a vehicle cooperates with another vehicle that is untrustworthy, that vehicle could provide an incorrect navigation route or cause a dangerous situation. This domain can be classified as trustworthiness from a collective point of view because the unmanned vehicles included in the CrowdNav-UV system evaluate trust with a common goal, which is a better traffic situation. Next, we use a reviewer verification service to show trust from an individual point of view. The reviewer verification service is a concept similar to an electronic assistant that selects and preferentially provides trustworthy reviews from among all reviews on a product or service that a user wants to purchase [48]. When a customer buys something in an online store, it is important to determine which of the numerous reviews are helpful and trustworthy ones. If that customer relies on untrustworthy reviews, this could lead to an unsatisfactory purchase or service experience. Because the evaluation and influence of a review differs from person to person, the reviewer verification service can be considered to be a domain for evaluating trust from an individual point of view.

In the case study design methodology, we need to define the study question from the research question and define the study proposition from the study question. The study proposition is divided into units of analysis to evaluate whether or not the proposition has been satisfied. Table 2 lists the research questions, study questions, and study propositions. The study propositions are composed of the general propositions and specific propositions. The specific propositions describe the detailed propositions derived from the general propositions.

Next, the units of analysis suggested for verifying each proposition are listed in Table 3. Some of them can be collected in each step of the proposed approach in the theoretical evaluation, and others must be collected from the survey of SMEs in the empirical evaluation. The description of why each unit of analysis can support the corresponding proposition is described as well. In the theoretical evaluation, we apply the proposed approach to two application domains and derive the artifacts using the proposed approach. In the empirical evaluation, SMEs compare the results before and after applying the proposed approach to the same domains, and we collect the data used for the units of analysis.

## 5. Theoretical Evaluation

In the theoretical evaluation, we considered two case studies to evaluate how the proposed approach could be applied to different application domains step by step by focusing on the generated models and artifacts as the units of analysis of the study proposition.

### 5.1. Domain 1: CrowdNav-UV

The first domain is the CrowdNav-UV system. As unmanned vehicles become more widespread, it will become possible for these vehicles in the road network to share various information with and provide various services to others, helping all vehicles on the roads obtain the optimal navigation results. In this process, there may be cases in which incorrect information is provided or information sharing is refused for personal benefit rather than maximizing the public interest of all users. Therefore, whenever information is required, it is important to choose a trustworthy cooperation partner to keep the real-time road network information useful. Based on this background, the following domain scenario is obtained [49]:

*While driving without driver intervention, a UV performs many instances of cooperation with other systems or vehicles and updates its road network information. When the destination information is entered into a UV, instructing it to move to a specific destination, it sets an initial route based on its network information and starts the journey. During the journey, various information is collected while cooperating with other trustworthy systems, and based on the collected information, the UV updates its road network information and determines a new optimal route. The optimal route is selected using a distance-priority method or time-priority method according to the driver’s preference. The UV travels to the destination while searching for and updating the optimal route based on real-time information according to the selected method.*

Based on this scenario, the representative requirement “drives to the destination” is derived, and the proposed approach is applied to this requirement. The first step of phase 1 is the requirements analysis, and the use case model listed in Table 4 is obtained.

In the second step, from the analyzed use case model, the system engineer derives the partial goal model. The first process in this step is the three-layer goal derivation. In the first layer, the use case goal layer, “the car drives to the destination without any accidents” statement of the *goal in context* element becomes the use case goal, and this represents the main objectives of the use case. The second layer is the actor goal layer, which represent the user’s behaviors in the *description* and *extension* elements. The goal instances are “the driver enters the destination,” “the driver selects the shortest route option,” and “the driver selects the fastest route option.” The last layer is the system behavior goal layer. This layer represents the system’s tasks in the description and extension elements, and there are five goal instances: “the car collects the traffic information,” “the car determines the shortest route,” “the car moves to the destination,” “the car determines the fastest route,” and “the car changes the route.” The derived goal instances are connected in a hierarchical relationship according to the layer hierarchy. All actions of the user are linked to the use case goal, and a series of system actions corresponding to the actions of the user are linked to each actor goal as a subgoal.

**Figure 4:**
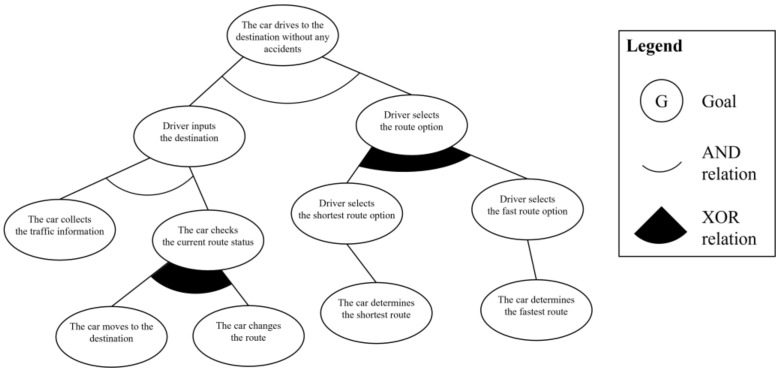
Partial goal model for “drives to the destination”.

The second process is defining the abstract goal to represent the points of variation in the scenario. In the given use case model, there are two such points: “the driver selects the route option” and “the car checks the current route status.” These points are considered as the abstract goal instances, and they are added between the goal instances, which are already connected. The third process is defining the additional relationships AND and XOR between goal instances. The AND relationship is added between the basic upper and lower hierarchical goal instances because the lower goal instances should be satisfied to achieve the upper goal instance. The XOR relationship is added between the abstract goal instances and its subgoals because only one of them should be satisfied. Figure 4 shows the derived partial goal model of the given scenario.

**Figure 5:**
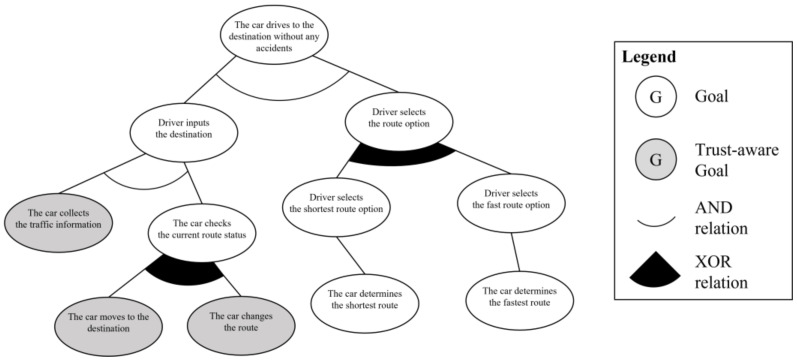
Trust-aware partial goal model for “drives to the destination”.

The next step is to analyze the trust-aware goal instance from the partial goal model. By applying the three criteria of the trust-requiring situation to the goal instances, the system engineer can verify which goal instances should be trust-aware. The first criterion is *informative*. The system engineer should check which goal instances require information by checking the required information element in the use case model. In the given scenario, there are three kinds of information: “traffic information,” “the positions of other cars,” and “emergency information.” The traffic information is used in “the car collects the traffic information” and “the car changes the route” goal instances to determine the route. The positions of the other cars and emergency information are used in “the car moves to the destination” goal instance to change the direction of travel or avoid an emergency situation. Three goal instances satisfy the first criterion and are passed to the second criterion, *interactive*. To evaluate the *interactive* criterion, the system engineer checks whether the source of the information must come from outside the system. In the scenario and goal instances, all information should come from outside the system for the better decision making; thus, all goal instances satisfy the second criterion. As the last task, the system engineer checks the *arbitrary* criterion. This is a process of confirming whether the cooperative target to obtain information is not a designated system but is randomly selected. If the system cooperates with the target system only, it does not need to check the trustworthiness of that system because it has already been verified. All three goal instances need to cooperate with randomly selected systems because it is necessary to cooperate with other vehicles currently on the road. Consequently, “the car collects the traffic information,” “the car changes the route,” and “the car moves to the destination” are newly defined as trust-aware goal instances because they satisfy all the criteria of a trust-requiring situation. Figure 5 shows the trust-aware partial goal model of “drives to the destination” requirement. The trust-aware goal instances are represented as gray circles.

**Figure 6:**
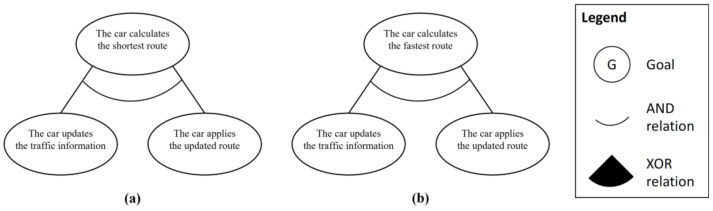
(**a**) Partial goal model for subordinate use case “calculate the shortest route” and (**b**) partial goal model for subordinate use case “calculate the fastest route”.

The last step of phase 1 is goal integration. To perform the goal integration in the given scenario, we additionally analyze the subordinate elements of this use case. There are two subordinate elements, “calculate the shortest route” and “calculate the fastest route,” as Figure 6 shows. Because this example is only used to show the integration process, each goal model has just two derived subgoal instances.

**Figure 7:**
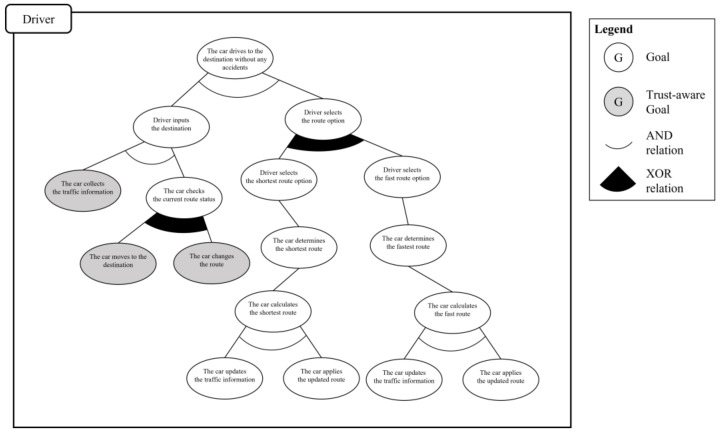
Integrated goal model for CrowdNav-UV.

In the goal integration process, there are three rules: (1) the *actor grouping rule*, (2) the *explicit integration rule*, and (3) the *implicit integration rule*. The first rule, the actor grouping rule, is used to make a group based on the main actor elements of the use case model. These three goal models have the same main actor elements with the analyzed use case model; thus, they are combined into the same actor group. The second rule is the explicit integration rule, which analyzes the parent–child relationship that appears clearly between the grouped goal models as the subordinates and the superordinate elements. The analyzed trust-aware partial goal model derived from the superordinate use case model is assigned to the upper layer, and the other three goal models derived from the subordinate use case models are assigned to the lower layer. The last rule, the implicit integration rule, analyzes the relevant parts in the scenarios of different use case models to derive detailed connection points between the goal models organized in a parent–child relationship. In the given scenario, “the car determines the shortest route” and “the car calculates the shortest route” are connected, and “the car determines the fastest route” and “the car calculates the fastest route” are also connected, because calculating a new route is required to determine a new route. Consequently, the derived trust-aware goal model is shown in Figure 7. Through the defined trust-aware system goal model, it is possible to understand the moments trust is important during the system development process, and as a result, develop a more trustworthy system.

**Figure 8:**
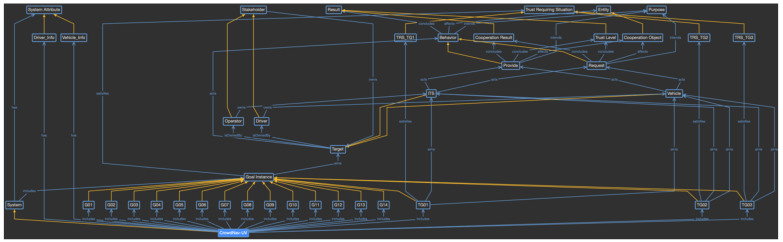
Domain-specific provenance model for CrowdNav-UV.

Phase 2 focuses on the process of defining and using the provenance model. The first step is to define the domain-specific provenance model as an ontology based on the proposed provenance meta-model. Using the provenance meta-model, the concepts of the ontology are analyzed in the scenario, and the classes and instances of the ontology are defined, as shown in Figure 8. In this figure, blue lines indicate object properties shared between classes and individuals, and yellow lines indicate individuals or subclasses of the class.

As Figure 8 shows in detail, CrowdNav-UV is defined as the individual of the system class. Its attributes are defined as system attribute concept classes, such as Driver_Info and Vehicle_Info. Because CrowdNav-UV consists of only one element, it becomes the instance. However, because the system attributes are defined in a variety of ways and can change over time, they are defined as the subclasses, and each event is accumulated as an individual of the classes. The goal instances defined in phase 1 are defined as the subclasses of the goal instance class, and whenever a goal instance is triggered, a new individual is created for that goal instance class. A subclass of the trust-requiring situation is also defined for the specific goal instance classes, which are represented as the trust-aware goal. This subclass is used to verify which goal instance is classified as a trust-aware goal instance. Next, in the target class, when the trust-aware goal instance is triggered, the targets to be evaluated are created as subclasses. In the given scenario, the vehicle or Intelligent Transport System (ITS) becomes the subclass of the target class. Each target is owned by a specific stakeholder. The stakeholder class also includes possible objects as subclasses. In this scenario, there are operator and driver subclasses. in the same way, the behavior class has provide and request subclasses, the entity class has the cooperation partner subclass, and the result class has cooperation result and trust level subclasses. The purpose class does not have any subclasses, and the individual is directly generated.

**Figure 9:**
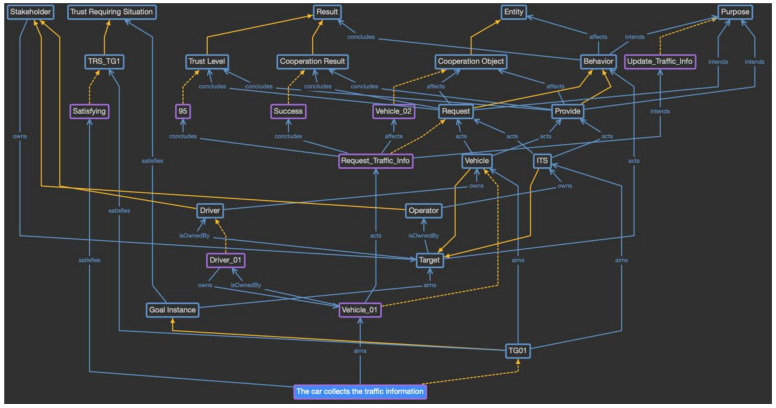
Provenance model for the specific event of Vehicle_01.

In this way, the domain-specific provenance model is defined by reflecting the characteristics of the CrowdNav-UV system. As the individuals of each class are created during the actual system operation, a knowledge base to evaluate trust is built and used for the evaluation. For example, when “the car collects the traffic information” goal is triggered and Vehicle_01 has been selected as the cooperation partner, the vehicle requests the provenance model of Vehicle_01, as shown in Figure 9. The purple boxes indicate the generated individuals, and the yellow dotted lines indicate a relationship between the class and individual. “The car collects the traffic information” goal satisfies the criteria for a trust requiring situation; thus, it is considered to be a trust-aware goal. Vehicle_01 requests traffic information from Vehicle_02, as represented by the behavior and cooperation classes, respectively. Vehicle_01 is owned by Driver_01, and the cooperation result was successful with a trust level of 95. Such provenance models are accumulated from the vehicle’s behaviors and used to evaluate trust.

Next, for the trust evaluation process of the system, a cooperation pattern is defined to increase the accuracy of trust evaluation by considering the characteristics of CrowdNav-UV. Various patterns can be analyzed according to specific domains. In this domain, two representative patterns that reflect the characteristics of trust from a collective point of view are described. First, it is necessary for all participants to share information to increase the public gain, but in the case of a system that only requests information for its own benefit, the trustworthiness of that system is judged to be low. Conversely, if the system shares a variety of information and the results are appropriate, the system can be viewed more positively, which can serve as an advantage in the trust evaluation process. These two patterns are analyzed as shown in Table 5. The consume-oriented pattern is a penalty type pattern. It uses the ratio of data requested to the total cooperation results of the target as the discriminant equation, and the influence equation determines the rate of trust reduction based on the discriminant equation and influence constants. Among the influence constants, α is the influence effect constant to determine how much influence the pattern has, and $\beta_{C}$ is the discriminant acceptance constant to determine whether the pattern is applied or not based on the result of the discriminant equation. These constants are defined based on the understanding of the domain by domain experts and software engineer with the heuristic analysis. In this case study, they are defined as 1.5 and 0.7, respectively. The provide-oriented pattern is an incentive type pattern. It uses the ratio of the data provided to the total cooperation results of the target as the discriminant equation, and the influence equation determines the trust increase rate based on the discriminant equation and the Influence constants. As same with the consume-oriented pattern, α is the influence effect constant and $\beta_{P}$ is the discriminant acceptance constant. They are also defined as 1.5 and 0.7, respectively, in this case study. That is, both patterns have the same influence on the trust evaluation result and the same threshold to determine whether the pattern is applied or not. These formulas are designed by domain experts with a heuristic approach based on domain knowledge. It this domain, the influence of the discriminant equation increases and decreases exponentially. Since the information provided by the vehicle changes from time to time, the influence needs to change rapidly over time; thus, the exponential function is used. The degree of increase and decrease is determined by influence constants. In addition, both patterns are only applied when the number of cooperation behaviors is greater than a pre-defined window size (W).

This makes it possible to identify the hidden intentions and manipulated results of the evaluation target in the trust evaluation process of the vehicle. In addition, it is possible to increase the trustworthiness of the group by excluding subjects that lower the trustworthiness of the group or by adding new trustworthy subjects into the group.

### 5.2. Domain 2: Reviewer Verification Service

The second case study is performed in the reviewer verification service from an individual point of view. Before buying a product or using a service, it is necessary to gather reviews about the product or service. Among the various reviews, it is important to determine appropriate reviews written by trustworthy reviewers. A reviewer verification service is a service that selects and provides trustworthy reviews to users. The following is the scenario of this domain [49].

*The reviewer verification service collects various reviews of the products or services desired by the user and selects trustworthy reviews to provide to the user. It is important to check the information about the reviewer who wrote the review. When a user adopts the reviewer verification service to purchase a product, other buyers’ reviews are collected from the various stores where they wrote the reviews. Then, the service selects trustworthy reviews from among the collected information and provides them to the user. In the process of selecting the reviews to be provided, the contents of the reviews and reviewers are verified to determine if they are trustworthy and meet the user’s preferences. If there are no reviews that can be recommended, the system can change the verification criteria to select other reviews. Finally, the user decides to purchase the product based on the collected trustworthy reviews.*

In this scenario, “provides the review information” is considered to be the representative requirement. It is analyzed as the use case model in the requirements analysis step of phase 1. Table 6 presents the details of the “provides the review information” use case model.

**Figure 10:**
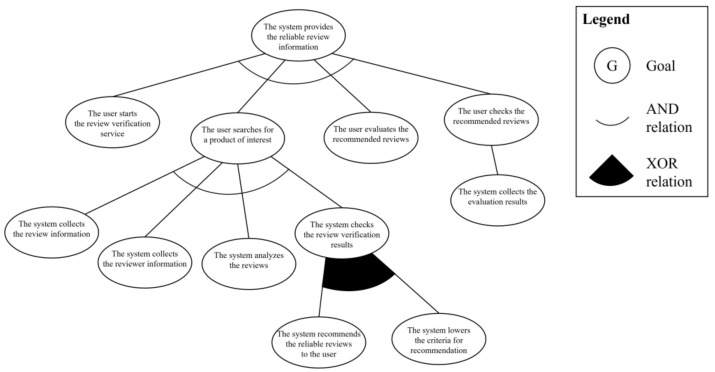
Partial goal model for “provides the review information”.

In the next step, the system engineer derives the partial goal model based on the analyzed use case model. First, the three-layer goal instances are derived. In the use case goal layer, “the system provides the reliable review information” is derived from the goal in the *context* element of the use case model. In the actor goal and system behavior goal layers, the user’s actions and system’s behaviors are separately derived in the description and extension elements of the use case model. The actor goal layer includes “the user starts the reviewer verification service,” “the user searches a product of interest,” “the user checks the recommended reviews,” and “the user evaluates the recommended reviews.” The system behavior layer has “The system collects the review information,” “The system collects the reviewer information,” “The system analyzes the reviews and the reviewer,” “The system recommends the reliable reviews to the user,” “The system collects the evaluation results,” and “The system lowers the criteria for recommendation.” The next step is to define the abstract goal instances to merge the variation points in the scenario. In this scenario, there is one variation point: “The system checks the reviews verification results.” As the next step, the goal instances are basically connected based on the layer hierarchy and extension relations with AND and XOR relationships. The derived partial goal model is shown in Figure 10.

**Figure 11:**
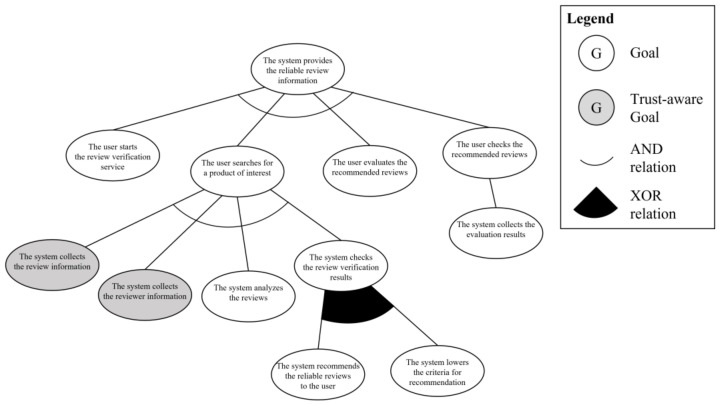
Trust-aware partial goal model for “provides the review information”.

Third step of phase 1 is the trust-aware goal analysis. To apply the *informative* criterion, the system engineer uses the required information elements in the use case model by analyzing at which stage each information is utilized in the scenario. The review and reviewer information are used in “the system collects the review information” and “the system collects the reviewer information,”, respectively. Therefore, these two goal instances satisfy the first criterion. Second criterion is *interactive*. Among the goal instances that satisfy the first criterion, all of the information should be gathered from other systems because the service only has the review information written by the current user. The last criterion is *arbitrary*. The review and reviewer information should be collected by cooperating with unknown systems and users because each bit of information is stored on the system of the reviewer of the products. Consequently, “the system collects the review information” and “the system collects the reviewer information” goal instances satisfy the criteria for a trust-requiring situation and become trust-aware goal instances. Figure 11 shows the trust-aware partial goal model of “provides the review information” requirements.

**Figure 12:**
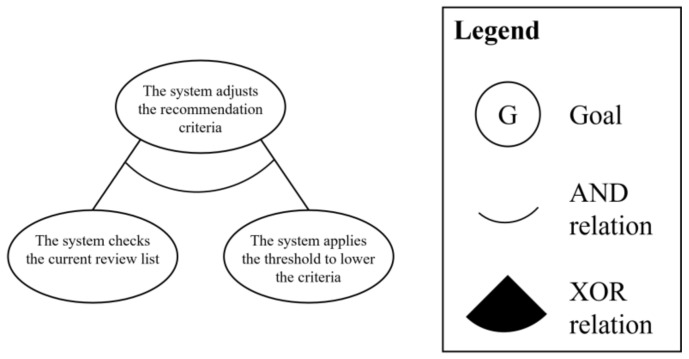
Partial goal model for the subordinate use case “adjusts the recommendation criteria”.

In this domain, as same in the previous domain, use cases in the subordinate element are simply analyzed as the partial goal models to show the process of performing the goal integration process. There is one subordinate element: “adjusts the recommendation criteria.” Figure 12 shows the analyzed partial goal model.

**Figure 13:**
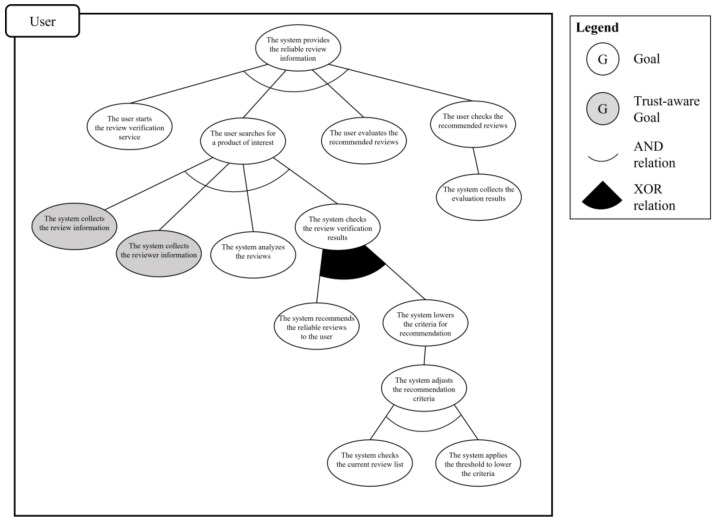
Integrated goal model for the review verification service.

First rule in the goal integration process is the actor grouping rule. In this scenario, all partial goal models have the same main actor; thus, they are classified into the same group. In the explicit integration rule, because “provides the review information” use case is a superordinate use case with respect to the other use cases, it is considered to belong to the upper layer and the other use cases are considered to belong to the lower layer. That is, the edge of the “provides the review information” goal model is connected to the top of the “the system adjusts the recommendation criteria” goal model. In the implicit integration rule, the system engineer clarifies the connecting point by analyzing the relationships in the scenario. In this scenario, “the system lowers the criteria for recommendation” is connected to “the system adjusts the recommendation criteria”. Consequently, the trust-aware system goal model shown in Figure 13 is derived. Using the defined trust-aware goal model in the system development process makes it possible to understand the moments when trust is important and develop a more trustworthy system.

**Figure 14:**
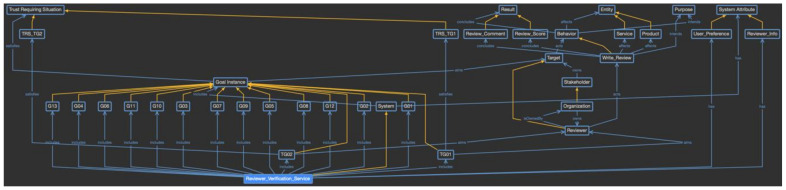
Domain-specific provenance model for review verification service.

Phase 2 is the process of defining and analyzing the provenance model as the trust evidence model, and the first step is to define the domain-specific provenance model as an ontology based on the proposed provenance meta-model. Using the defined meta-model, the concepts are analyzed in the scenario and the classes and individuals of the ontology are defined, as shown in Figure 14.

As the detail in Figure 14 shows, the reviewer verification service is defined as the individual of the system class, and its attributes are defined as the classes of the system attribute concept as Reviewer_Info and User_Preference. Because the reviewer verification service consists of only one element, it directly becomes the instance of the system class. On the contrary, the system attributes are diverse and change over time. Thus, they are defined as the subclasses, and each event is accumulated as an individual for each class. The goal instances defined in phase 1 are defined as the subclasses of the goal instance concept, and whenever a goal instance is triggered, a new individual is created for that goal instance class. The subclasses of the trust-requiring situation are also defined for the specific goal instance classes, which are represented as the trust-aware goal. Next, in the target class, when the trust-aware goal instance is triggered, the targets to be evaluated are created as subclasses. In the given scenario, the reviewer becomes the subclass of the target class. The target is owned by the specific stakeholder, which is represented as a subclass of the stakeholder class. In the scenario, the organization class is created as a subclass of the stakeholder class. In the same way, the behavior class has Write_Review subclasses, the entity class has product and service subclasses, which are the objectives for the review, and the result class has review comment and review score subclasses, which are the outcome of writing a review. The purpose class does not have a subclass, and the individual is directly generated.

**Figure 15:**
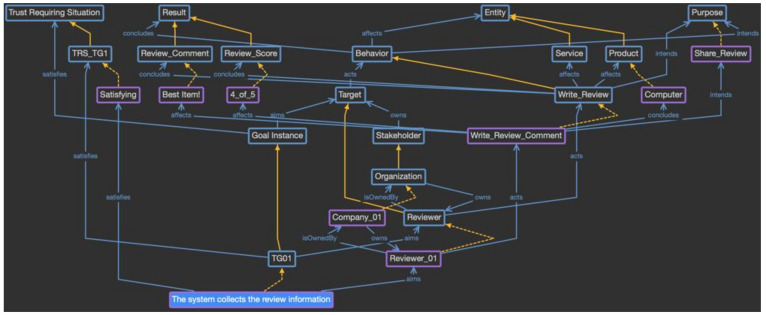
Provenance model for the specific event of Reviewer_01.

In this way, the domain-specific provenance model is defined by reflecting the characteristics of the reviewer verification service. Moreover, because instances suitable for each class are created during the actual system operation, a knowledge base to evaluate trust is built and used for the trust evaluation. For example, Figure 15 shows the situation when “the system collects the review information” goal has been triggered, and Reviewer_01 has been selected as the target to be evaluated. The purple box indicates the generated individuals, and the yellow dotted line indicates the relationship between the class and individual. Because this goal satisfies the criteria for a trust-requiring situation, it is considered to be a trust-aware goal, and other information is collected. Reviewer_01 belongs to Company_01, and this reviewer performs a Write_Review_Comment behavior for the computer product as an entity under the Share_Review purpose. The result of this behavior includes the review comment with the score. This provenance model will be used to evaluate the review’s trustworthiness by tracking the stakeholder and result values.

Next, for the trust evaluation process of the system, the cooperation pattern is defined for a more accurate trust evaluation by considering the characteristics of the reviewer verification service from the point of view of an individual. Considering the characteristics of the domain in the trust evaluation process, one person can leave the negative reviews for all products. Conversely, it is possible to manipulate the reviews by giving only positive reviews for all products. Because these behaviors follow a pattern, we can claim that the first pattern is an abusing pattern, and the second pattern is an overusing pattern. Both are considered to be a penalty type pattern because these kinds of behavior decrease the reviewer’s trust. The discriminant equation of the abusing pattern uses the ratio of the number of negative reviews to the number of total reviews, and it is used in the influence equation with the influence constants γ and $\beta_{A}$. The discriminant of the second pattern uses the ratio of the number of positive reviews to the number of total reviews, and it is also used in the influence equation with the influence constants γ and $\beta_{O}$. Both patterns are only applied when the number of total reviews is greater than the predefined window size W. For the simplified case study, we also define the influence effect constant γ with 1.5 and the discriminant acceptance constants $\beta_{A}$ and $\beta_{O}$ with 0.7, respectively. These constants are defined based on the understanding of the target domain and characteristics of the analyzed pattern. These formulas are also derived from domain knowledge by domain experts with a heuristic approach. In this domain, the influence of the discriminant equation increases and decreases exponentially with Euler’s number. Because review products rarely change over time, Euler’s number can be used to effectively represent the natural increase and decrease of values used for the trust evaluation of review contents. The degree of increase and decrease is determined by influence constants. Table 7 summarizes the cooperation patterns in this domain.

This makes it possible to identify any hidden intentions and manipulated results of the evaluation target in the process of evaluating the trustworthiness of the review. In addition, because trustworthiness can be evaluated using individual standards for trust, different results can be derived depending on who is being evaluated, even for the same system.

## 6. Empirical Evaluation

The main purpose of the empirical evaluation is to verify the feasibility and assess the effectiveness and efficiency of the proposed approach. In the empirical evaluation, we conducted a comparison experiment of the legacy and proposed approaches with three SMEs with 2 to 10+ years of experience in software engineering.

The empirical evaluation process was as follows:

1. Explaining the background information: Because some of participants might not have been familiar with SASs or trust, we briefly explained the background knowledge to the participants.
2. Introducing the application domain: We introduced the application domain and the representative scenario that the participants were asked to analyze.
3. Applying the legacy approach to analyze the trust-aware requirements: Using the participants’ knowledge about requirements analysis, the scenario was analyzed, and the trust-aware requirements were obtained.
4. Introducing the proposed approach: We explain the proposed approach along with the generated models and artifacts step by step.
5. Applying the proposed approach to analyze the trust-aware requirements: Using the proposed approach, the participants analyzed the trust-aware requirements of the scenario in similar application domain.
6. Surveying the participants: We asked the users some questions about their experiences and impressions of the process of the requirements analysis.

By analyzing similar application domain using both the legacy and proposed approaches, we were able to compare the generated models and artifacts. This can help us determine the advantages and disadvantages of the proposed approach. In addition, through the survey, we can verify the effectiveness and efficiency of the proposed approach.

## 7. Evaluation Result

This section presents an analysis of the results of the theoretical and empirical evaluations in terms of the study questions and propositions. In the results of the theoretical evaluation, we captured evidence to support that the study propositions have been satisfied, as the results in Table 8 reveal. The proposed approach helps the analysis of trust-aware requirements. The study proposition can be satisfied by the evidence obtained in each step or the study question. The evidence was captured in specific steps in the framework. In both application domains, there are common-structured and domain-specific models. For the common-structured model, the goal model is driven based on the scenario of each domain. By contrast, for the domain-specific model, the provenance model has different elements for each application domain. Even though both provenance models were generated based on the provenance-meta-model, the CrowdNav-UV system has vehicle-related subclasses, such as driver and vehicle. By contrast, the reviewer verification system has review-related subclasses, such as reviewer and user preference.

From the results of the empirical evaluation, we can determine how the proposed approach helps the system engineer analyze the trust-aware requirements and design the domain-specific provenance model for the trust evaluation. We conducted the case study with three SMEs to compare the legacy approach with the proposed approach. After the case study, which included two different application domains, we examine the analyzed artifacts obtained from the case study and investigated what the SMES felt during the experiment. Table 9 shows the results of the experiments.

UA12 is the number of goal instances. It is important to determine how many goal instances can be analyzed with the proposed approach. We compared how many goal instances were analyzed by each SME using the legacy requirement engineering and the proposed approach. As Table 9 reveals, all SMEs analyzed more goal instances using the proposed approach in both domain applications. The number where the arrow starts is the number of analyzed goal instances from the legacy approach and the number the arrow points to is the number of analyzed goal instances from the proposed approach. Even though the number of analyzed goal instances differs depending on the proficiency of each SME at the goal modeling, defining goal modeling using the proposed approach tends to lead to the analysis of more goal instances. UA13 is the usability of deriving goal models. We provide a process for deriving the goal model from the user requirements. This process should help the user derive the goal model, but we must investigate how helpful this process was. All SMEs answered that the proposed approach was helpful for deriving the appropriate goal instances with the exception of SME 1 for domain 1. The reason why SME 1 gave UA13 a score of 3 for domain 1 is that the results obtained with and without the proposed approach were almost the same, even though the steps of the proposed approach were more complicated. In fact, when they used the proposed method, we ask users to perform slightly more complex and systematic steps to derive the goal model. It is recognized that this can have a negative impact in some cases. However, for domain 2, SME 1 felt that the proposed approach was more effective and usable than the legacy goal modeling approach to derive additional goal instances. The number of trust-aware goal instances is captured as UA14. After explaining the concept of trust in the requirement engineering process, the SMEs were asked to analyze the trust-aware goal instances with and without the proposed approach. The number where the arrow starts is the number of the trust-aware goal instances without the proposed approach and the number the arrow points to is the number of the trust-aware goal instance with the proposed approach. The results are overall the same with and without the proposed approach. This indicates that the proposed approach can properly derive trust goal instances at a commonsense level. UA15 represents the usability for analyzing the trust-aware goal model. We provided the criteria for identifying general goal instances that are trust-aware goal instances, but we need to investigate how helpful the proposed criteria were for identifying trust-requiring situations. The results show that the proposed approach almost always helped SMEs analyze trust-aware goal instances. Consequently, the goal models derived by the legacy and proposed approaches should be compared in terms of how satisfactorily the user’s requirements were captured and expressed. This is captured as satisfaction with the derived goal model (UA16), and all SMEs gave a high satisfaction score to the goal model derived from the proposed approach, as Table 9 shows.

UA17 is the number of provenance model classes. The provenance model is one of the most important artifacts for evaluating the system trust because it is used as the evidence of trust. We can compare the number of provenance model classes that the SMEs analyzed with the result of the theoretical evaluation, excluding the common meta-model instances. The numerator is the number of provenance model classes that the SMEs analyzed, and the denominator is the number of provenance model classes analyzed from the theoretical evaluation. This model leads to significant differences depending on the SME’s thoughts and characteristics. As Table 9 shows, SME 1 defined fewer provenance model classes than the theoretical evaluation result. This is not an incorrect answer, but a different point of view for analyzing system trust. Using their provenance model, they can define their trust according to the evidence. In addition, because the given scenario is limited, the results are also extremely restricted. By contrast, SME 2 defined more provenance model classes than the theoretical evaluation result. This indicates that SME 2 has more background knowledge about defining the provenance model for the trust evaluation in domain 2. We next consider UA18, the effectiveness of the meta-model. When the SMEs analyzed the provenance model, the proposed meta-model was used as the basis. We need to investigate how helpful the proposed meta-model was for defining the domain-specific provenance model. The SMEs mostly gave the meta-model a high score, which indicates that the meta-model was helpful and reasonable for modelling the new type of trust evidence models.

UA19 is the usability of evaluating trust from the provenance model. In the proposed approach, the SMEs used the provenance model to evaluate the system trust, and in this specific scenario, we need to investigate whether the evaluated trust value is reasonable. We briefly described the example scenario to the SMEs, who evaluated the trust value using the legacy and proposed approaches. The legacy approach uses an evaluation based on a simple rating value. The scenario includes both trustworthy and untrustworthy candidates with malicious intentions. In this situation, we asked the SMEs if the evaluated results were appropriate for the given scenario. They answered that the trust value from the proposed approach yields more reasonable and usable results. However, this trust value does not consider the cooperation patterns, which are the most valuable aspect of the proposed approach. Thus, we examined the number of analyzed cooperation patterns and the satisfaction with the application of the cooperation patterns (UA20 and UA21, respectively). The cooperation pattern is a key element for analyzing the hidden intentions of a system, and it is important that the appropriate pattern is defined. We can compare the number of analyzed cooperation patterns obtained by the SMEs with the results of the theoretical evaluation. The numerator is the number of analyzed cooperation patterns suggested by the SMEs, and the denominator is the number of analyzed cooperation patterns obtained from the theoretical evaluation. Most SMEs analyzed the same number of patterns as the theoretical evaluation result. Even though the application domain was explained in detail, they focused on the given scenario during the evaluation process and analyzed the specific pattern described in the scenario. More important than the number of analyzed pattern is how influential the patterns were. That is, even though the cooperation patterns were analyzed well, it is more important how positively they affected the trust evaluation results. In each specific scenario, we compared the trust evaluation results obtained with and without the cooperation patterns. As Table 9 reveals, all SMEs said that the trust evaluation with the cooperation patterns was better than the trust evaluation without the cooperation patterns. This indicates that the cooperation pattern is effective for determining the malicious intention behind behaviors. Consequently, the trust values were calculated for each cooperation candidate with the legacy trust evaluation method and the proposed approach. By considering the given scenario, the SMEs compared the results and discussed which one was more appropriate for the current scenario (UA22). Most of them gave a higher score to the results of the proposed approach in both application domains. This is a result of the reasonable trust evidence model, which represents the various facets of the system, and the diagnosis of the malicious intention behind the normal behaviors.

Consequently, as the results in Table 10 reveal, all study questions, general propositions, and specific propositions are satisfied by the units of analysis captured in the case study. The captured information was an objective evaluation of the empirical evaluation results or was obtained by comparing the artifacts generated before and after the application of the proposed approach by three SMEs. On the basis of this result, we claim that the proposed approach is appropriate for analyzing and modeling the trust-aware requirements and the trust evidence model as the provenance model.

## 8. Conclusions and Future Work

In this study, we proposed a framework for considering and evaluating trust in the process of analyzing the requirements of SASs. The proposed framework consists of: (1) a process that can analyze trust-aware requirements by analyzing the characteristics of trust and (2) a provenance model as a trust evidence model with a trust evaluation method for using it. To derive a trust-aware goal model, the process of deriving a developer-friendly goal model from a user-friendly use case model is first proposed. Next, using criteria for trust-requiring situations, the trust-aware goal instances that need to be considered for trust are determined, and finally, the derived models are combined. Through this proposed process, it is possible to analyze how trust can be considered when analyzing system requirements. In addition, the new trust evidence model uses a new concept and provenance to verify trust that has been gradually accumulated rather than simply utilizing the fragmentary observations of the present. Using this approach, more meaningful trust evaluation results are derived by analyzing the cooperation patterns that can reveal the meaning of hidden intentions and actions of a system. In the case study, we demonstrated the proposed approach using two different evaluation processes in two application domains: the CrowdNav-UV system and a reviewer verification service. In the theoretical evaluation, we checked its ability to address the given research questions. In the empirical evaluation, we conducted a survey to investigate the usability of the proposed approach for three SMEs. Consequently, the observed evidence in the case study was used to prove that the proposed approach has the capability to handle the research problems.

As future work, we plan to further develop the algorithm and process for evaluating trust using the provenance model. The proposed method has a limitation in that sufficient provenance models are required for the appropriate trust evaluation. To address this, we plan to impose additional randomness in the process of selecting a trustworthy target by combining PageRank algorithms. In addition, the current algorithm requires domain-specific knowledge to specialize the equation for the target domain. To address this limitation, we should suggest the guideline to derive a domain-specific algorithm by using the analyzed domain-specific provenance model. We also plan to analyze the characteristics of trust more effectively from collective and individual points of view and subsequently apply them to the process of deriving the provenance model and trustworthiness evaluation. It will be helpful for system engineers when designing the template of the provenance model or emphasizing the characteristics of each point of view. Furthermore, the scope of this research is focusing on how trust can be modeled and evaluated; thus, after evaluating trust, the behavior of SAS is not considered. As the next research, we can analyze and provide the whole process of the trust-aware self-adaptation as well.

## Figures and Tables

**Table 1:** Modified use case template.

Element	Description
Use Case Name	Unique use case name
Goal in Context	General goal statement of the use case
Precondition	Prerequisite to be satisfied before starting the use case
End Condition	Closing statement after the success or failure of the use case
Primary Actor	Main stakeholder of the use case
Trigger	The action upon the system that initiates the use case
Description	Success scenario to describe interaction between the actor and system
Extension	Extended actions from the success scenario
Required Information	Information needed to complete the specific step, where it is assumed that such information exists
Superordinates	Names of the use cases that include this one (optional)
Subordinates	Names of the subuse cases (optional)

**Table 2:** Research questions, study questions, general propositions, and specific propositions for the case study.

Research Question	Study Question	General Proposition	Specific Proposition
RQ 1. What is trust and what are the conditions under which trust is required?	SQ 1. How can the proposed approach analyze the trust-aware requirements?	GP 1. The proposed approach can analyze the requirements from the user.	SP 1.1. The proposed approach provides a method for analyzing the requirements from the user and derives the goal model.
		GP 2. The proposed approach can identify the trust-related elements in the requirements.	SP 2.1. The proposed approach converts the general goal instance into a trust-aware goal instance based on the trust-requiring situation.
RQ 2. What are the necessary grounds for determining a trustworthy system, and how can we model those grounds?	SQ 2. Why can the provenance model be used for the trust evidence model?	GP 3. The provenance model can represent the trust-related information for a specific domain.	SP 3.1. The proposed approach helps the system engineer to understand what is required for evaluating trust based on the provenance meta-model.
			SP 3.2. The proposed approach helps the system engineer to define the domain-specific provenance model based on the provenance meta-model.
	SQ 3. How can the provenance model be used to evaluate the system trustworthiness?	GP 4. The provenance model can be used for evaluating the system trust by considering the various aspects of trust.	SP 4.1. The proposed approach provides the provenance-based trust evaluation algorithms to consider trust from a fragmentary point of view.
			SP 4.2. The proposed approach provides the cooperation patterns needed to analyze the system trust from a complex point of view.

**Table 3:** Units of analysis to be obtained from the evaluation process.

Units of Analysis			
Theoretical Evidence		Empirical Evidence	
Code	Evidence Name	Code	Evidence Name
UA01	Use Case Model	UA12	Number of Goal Instances
UA02	Goal Derivation Processes	UA13	Usability of Deriving the Goal Model
UA03	Partial Goal Model	UA14	Number of Trust-Aware Goal Instances
UA04	Trust-Requiring Situation	UA15	Usability for Analyzing the Trust-Aware Goal Model
UA05	Trust-Aware Goal Model	UA16	Satisfaction with the Derived Goal Model
UA06	Integration Rule	UA17	Number of Provenance Model Classes
UA07	Integrated Goal Model	UA18	Effectiveness of the Meta-model
UA08	Provenance-Meta-Model	UA19	Usability of Evaluating Trust from the Provenance Model
UA09	Domain-Specific Provenance Model	UA20	Number of Analyzed Cooperation Patterns
UA10	Provenance-Based Trust Algorithm	UA21	Satisfaction with the Application of the Cooperation Patterns
UA11	Cooperation Pattern	UA22	Comparison with the Legacy Trust Evaluation Method

**Table 4:** Use case text for “drives to the destination”.

Element	Description
Use Case Name	Drives to the destination
Goal in Context	The car drives to the destination without any accidents
Precondition	The driver has the destination
End Condition	The car arrives at the destination
Primary Actor	The driver
Trigger	The driver turns on the navigation mode
Description	The driver inputs the destination The car collects the traffic information. Driver selects the shortest route option. The car determines the shortest route. The car moves to the destination. Repeat steps 3–5 until the car arrives at the destination.
Extension	3a. If the driver wants the fastest route option: 3a-1. the driver selects the fastest route option 3a-2. the car determines the fastest route 5a. If the car encounters obstacles: 5a-1. the car changes the route.
Required Information	- The traffic information - The position of the other cars - The emergency information
Superordinate	Drives to the destination
Subordinate	The car drives to the destination without any accidents.

**Table 5:** Cooperation patterns for CrowdNav-UV.

Name	Type	Discriminant Equation	Influence Equation
Consume oriented	Penalty	$D_{C}=\frac{N_{\mathrm{request}}}{N_{\mathrm{total}.}}$	${\mathrm{IE}}_{\mathrm{consume}}=\left\{\begin{array}{c} 1-{\left(\frac{D_{C}}{2}\right)}^{\alpha }\left(D_{C}\geq \beta_{C},\;N_{\mathrm{total}}\geq W\right)\\ 1\;\left(\mathrm{others}\right) \end{array}\right.$
Provide oriented	Incentive	$D_{P}=\frac{N_{\mathrm{provide}}}{N_{\mathrm{total}}}$	${\mathrm{IE}}_{\mathrm{provide}}=\left\{\begin{array}{c} 1+{\left(\frac{D_{P}}{2}\right)}^{\alpha }\left(D_{P}\geq \beta_{P},\;N_{\mathrm{total}}\geq W\right)\\ 1\;\left(\mathrm{others}\right) \end{array}\right.$

**Table 6:** Use case text for “provides the review information”.

Element	Description
Use Case Name	Provides the review information
Goal in Context	The system provides reliable review information
Precondition	The user has a wishlist
End Condition	The user stops shopping
Primary Actor	The user
Trigger	The user starts the reviewer verification service. The user searches for an item.
Description	The user starts the reviewer verification service. The user searches for a product of interest. The system collects the review information. The system collects the reviewer information. The system analyzes the reviews and reviewer. The system recommends reliable reviews to the user. The user checks the recommended reviews. The user evaluates the recommended reviews. The system collects the evaluation results.
Extension	6a. There is no review that can be recommended. 6a-1. The system lowers the criteria for recommendation.
Required Information	- Review information - Reviewer information
Superordinate	None
Subordinate	Adjusts the recommendation criteria

**Table 7:** Cooperation patterns for the review verification service.

Name	Type	Discriminant Equation	Influence Equation
Abusing	Penalty	$D_{A}=\frac{N_{\mathrm{negative}}}{N_{\mathrm{total}}}$	${\mathrm{IE}}_{\mathrm{abusing}}=\left\{\begin{array}{c} e^{-{\gamma D}_{A}}\left(D_{A}\geq \beta_{A},\;N_{\mathrm{total}}\geq W\right)\\ 1\;\left(\mathrm{others}\right) \end{array}\right.$
Overusing	Penalty	$D_{O}=\frac{N_{\mathrm{positive}}}{N_{\mathrm{total}}}$	${\mathrm{IE}}_{\mathrm{overusing}}=\left\{\begin{array}{c} e^{-{\gamma D}_{O}}\left(D_{O}\geq \beta_{O},{\;N}_{\mathrm{total}}\geq W\right)\\ 1\;\left(\mathrm{others}\right) \end{array}\right.$

**Table 8:** Experimental results of the theoretical evaluation and supported propositions.

Study Question	Step in Framework	Captured Evidence	Supported Proposition
SQ 1	Phase 1—Step 1 Requirements Analysis	**UA01: Use Case Model:** It helps the system engineer to understand what the user wants and derive the requirements with a user-friendly approach It also contains the scenario that the system engineer expects between the system and user and is used for deriving the goal instances.	GP 1 SP 1.1
SQ 1	Phase 1—Step 2 Partial Goal Model Analysis	**UA02: Goal Derivation Processes:** To derive the goal model from the use case model, we suggest three goal derivation processes. The user can analyze which scenario elements should be the goal instances. In addition, the elements are simply connected using a hierarchical relationship.	GP 1 SP 1.1
		**UA03: Partial Goal Model:** The partial goal model represents the goal instances with respect to the relationship by focusing on the specific function. This helps the system engineer to translate the user-friendly written requirements into engineer-friendly written requirements.	
SQ 1	Phase 1—Step 3 Trust-Aware Goal Analysis	**UA04: Trust-Requiring Situation:** This helps the user analyze and interpret which goal instance should be trust-aware by analyzing the characteristics of trust. There are three criteria that must be checked step by step, but this is slightly open to human interpretation, which means the results can differ depending on who analyzes and applies the criteria.	GP 2 SP 2.1
		**UA05: Trust-Aware Goal Model:** This is the goal model containing the trust-aware goal instances. Thus, it helps the system engineer consider the trust-aware elements during the system development process with the specific evidence and criteria.	
SQ 1	Phase 1—Step 4 Goal Integration	**UA06: Integration Rule:** Because the goal models are derived from the specific functions, there is a set of goal models. To integrate them into the system goal model, we provide three integration rules using the certain elements in the use case model.	GP 1 SP 1.1
		**UA07: Integrated Goal Model:** The partially derived goal models are integrated into the system goal model. These models is grouped based on the main actor, and the vertical/horizontal relationship are derived based on the scenario and specific elements in the use case model.	
SQ 2	Phase 2—Step 1 Domain-Specific Provenance Model Analysis	**UA08: Provenance-Meta-Model:** By analyzing the definition of provenance, we designed a provenance meta-model to define the provenance model for the specific domain. It can convert the provenance model into an ontological model and helps the system infer the trust-related information.	GP 3 SP 3.1 SP 3.2
		**UA09: Domain-Specific Provenance Model:** Using the provenance-meta-model, the user can design a provenance model for a specific domain. During system runtime, the accumulated data are collected and used to evaluate the system’s trust. However, this is slightly based on human interpretation, which means the model can differ depending on who analyzed and designed the model.	
SQ 3	Phase 2—Step 2 Provenance-Based Trust Evaluation	**UA10: Provenance-Based Trust Algorithm:** We propose a simple algorithm considering not only single behaviors, but also the hidden intentions behind the accumulated behaviors. Depending on the domain, the system engineer can modify the factors in the algorithm.	GP 4 SP 4.1
SQ 3	Phase 2—Step 3 Cooperation Pattern Analysis	**UA11: Cooperation Pattern:** We provide a template for defining the cooperation pattern for the target domain. This helps the system engineer consider the hidden rationale behind the system behaviors. It is important to discover unknown malicious systems.	GP 4 SP 4.2

**Table 9:** Experiments result of the empirical evaluation and the supported proposition.

Study Question	Captured Evidence	Experimental Results						Supported Proposition
		SME 1		SME 2		SME 3		
		Domain 1	Domain 2	Domain 1	Domain 2	Domain 1	Domain 2	
SQ 1	UA12	14 -> 15	11 -> 19	15 -> 22	9 -> 15	5 -> 11	3 -> 8	GP 1 SP 1.1
SQ 1	UA13	3	4	5	5	5	4	GP 1 SP 1.1
SQ 1	UA14	1 -> 1	2 -> 2	1 -> 2	2 -> 3	2 -> 2	1 -> 1	GP 2 SP 2.1
SQ 1	UA15	5	5	5	5	3	4	GP 2 SP 2.1
SQ 1	UA16	4	4	5	5	5	3	GP 1 GP 2
SQ 2	UA17	6/12	6/10	12/12	11/10	12/12	10/10	GP 3 SP 3.2
SQ 2	UA18	5	4	5	5	3	5	GP 3 SP 3.1
SQ 3	UA19	4	4	5	5	4	4	GP 4 SP 4.1
SQ 3	UA20	2/2	2/2	2/2	2/2	1/2	2/2	GP 4 SP 4.2
SQ 3	UA21	5	5	5	5	5	5	GP 4 SP 4.2
SQ 3	UA22	4	5	5	5	3	5	GP 4 SP 4.1 SP 4.2

**Table 10:** Corresponding units of analysis for the study questions and research propositions.

Study Question	General Proposition	Specific Proposition	Corresponding Units of Analysis
SQ1	GP1	SP1.1	UA01, UA02, UA03, UA06, UA07, UA12, UA13, UA16
	GP2	SP2.1	UA04, UA05, UA14, UA15, UA16
SQ2	GP3	SP3.1	UA08, UA09, UA18
		SP3.2	UA08, UA09, UA17
SQ3	GP4	SP4.1	UA10, UA19, UA22
		SP4.2	UA11, UA20, UA21, UA22

## Data Availability

No new data were created or analyzed in this study. Data sharing is not applicable to this article.
